# Receptive field structures for two celestial compass cues at the input stage of the central complex in the locust brain

**DOI:** 10.1242/jeb.243858

**Published:** 2022-02-23

**Authors:** Naomi Takahashi, Frederick Zittrell, Ronja Hensgen, Uwe Homberg

**Affiliations:** ^1^ Department of Biology, Animal Physiology, Philipps-Universität Marburg, 35032 Marburg, Germany; ^2^ Center for Mind, Brain and Behavior (CMBB), University of Marburg and Justus Liebig University Giessen, 35032 Marburg, Germany

**Keywords:** Sun compass orientation, Insect brain, Central complex, Polarization vision, Desert locust

## Abstract

Successful navigation depends on an animal's ability to perceive its spatial orientation relative to visual surroundings. Heading direction in insects is represented in the central complex (CX), a navigation center in the brain, to generate steering commands. In insects that navigate relative to sky compass signals, CX neurons are tuned to celestial cues indicating the location of the sun. The desert locust CX contains a compass-like representation of two related celestial cues: the direction of unpolarized direct sunlight and the pattern of polarized light, which depends on the sun position. Whether congruent tuning to these two compass cues emerges within the CX network or is inherited from CX input neurons is unclear. To address this question, we intracellularly recorded from GABA-immunoreactive TL neurons, which are input elements to the locust CX (corresponding to R neurons in *Drosophila*), while applying visual stimuli simulating unpolarized sunlight and polarized light across the hemisphere above the animal. We show that TL neurons have large receptive fields for both types of stimuli. However, faithful integration of polarization angles across the dorsal hemisphere, or matched-filter ability to encode particular sun positions, was found in only two out of 22 recordings. Those two neurons also showed a good match in sun position coding through polarized and unpolarized light signaling, whereas 20 neurons showed substantial mismatch in signaling of the two compass cues. The data, therefore, suggest that considerable refinement of azimuth coding based on sky compass signals occurs at the synapses from TL neurons to postsynaptic CX compass neurons.

## Teaser

**Summary:** GABAergic input neurons to the locust sky compass detect sky polarization and sun position but show poor matched-filter performance, suggesting that faithful heading direction signaling emerges within the central complex.

## INTRODUCTION

Spatial orientation relative to visual surroundings is a crucial ability for successful navigation. Neurons representing an animal's orientation, such as head direction cells in the rat (Taube et al., 1990a,b), have been intensely studied (Cullen and Taube, 2017). Theoretical studies have proposed recurrent network models called ring attractors to explain neuronal population dynamics of heading representation (Knierim and Zhang, 2012; Skaggs et al., 1995). Theoretical and experimental data suggested that heading-direction systems are driven by internally generated self-motion cues, but most networks also use external sensory cues, such as visual landmarks, for feedback control.

Insects also show physiological signatures of heading-direction coding to visual references, enabling the characterization of ring attractor elements in biological circuits that consist of a much smaller number of neurons than mammalian systems (Green and Maimon, 2018; Turner-Evans et al., 2020). Heading direction is represented in the central complex (CX), a navigation center of the insect brain, to generate steering commands to a navigational goal. The CX is a group of midline-spanning neuropils consisting of the protocerebral bridge (PB), the upper and the lower divisions of the central body (CBU and CBL, also termed fan-shaped body and ellipsoid body), and the paired noduli. These neuropils are subdivided into vertical slices and horizontal layers by neuronal projection patterns (Heinze and Homberg, 2008; Hulse et al., 2021; Wolff et al., 2015). In *Drosophila*, heading direction is represented as a localized bump of population activity in the so-called E-PG neurons of a ring attractor (Green et al., 2017; Seelig and Jayaraman, 2015). E-PG neurons are topographically arranged in the slices of the CBL and PB. The activity bump moves to neighboring slices when the fly turns clockwise or counterclockwise, and optogenetic manipulation of the bump position leads to flight orientation shifts following the bump (Kim et al., 2019). This heading representation works in darkness but more reliably when a visual cue is available (Turner-Evans et al., 2020).

**Fig. 1.**
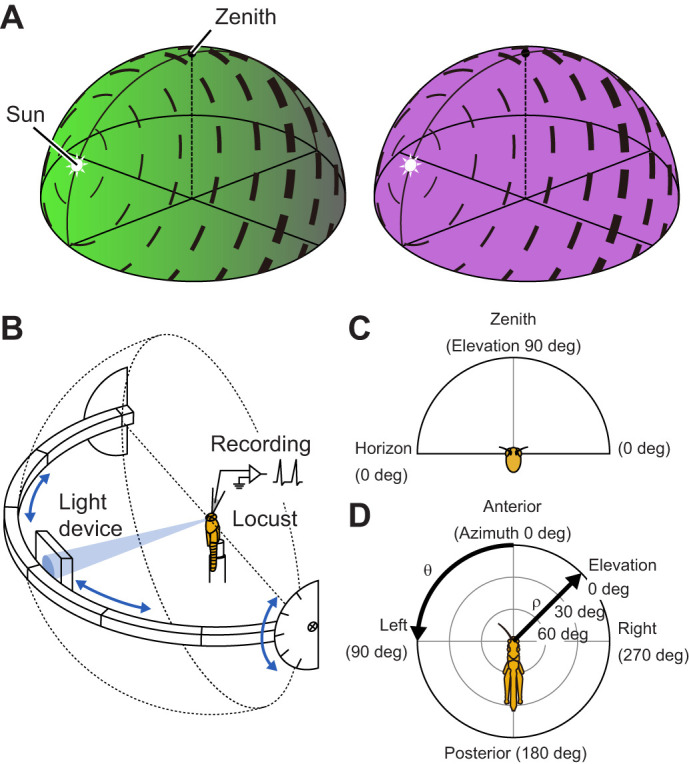
**Celestial compass cues and visual stimulation.** (A) Schematic illustration of the polarization pattern and color gradient in the sky. The AoP (black bar orientation) is arranged tangentially along concentric circles around the sun. The degree of polarization (black bar thickness) reaches its maximum at a given point of the sky when the great-circle distance is 90 deg from the sun. The chromatic gradient is the product of a gradient of long-wavelength light intensity (left, green) and a uniform distribution of short-wavelength light (right, UV). (B) Stimulus setup modified from Bech et al. (2014) and Zittrell et al. (2020). The light stimulus could be switched between polarized blue, unpolarized green and unpolarized blue during recording. After stimulation from the animal's zenith, the light device was shifted along the perimeter that could be tilted as indicated by blue arrows. It allowed stimulating the animal from directions in the entire dorsal visual field. (C) Frontal view of the spherical dorsal visual field of the animal. (D) Top view of the spherical dorsal visual field flattened on a polar-coordinate grid. The radius (ρ) is defined as 1–elevation/90 deg (0≤ρ≤1) and the angle (θ) equals the spherical azimuth (0 deg≤θ<360 deg). The elevation and azimuth are indicated relative to the animal's head.

In several insects, many neurons of the CX are tuned to visual stimuli simulating celestial cues (el Jundi et al., 2015; Hardcastle et al., 2021; Heinze and Homberg, 2007; Heinze and Reppert, 2011). Celestial cues are related to the location of the sun and include the direction of unpolarized direct sunlight as well as the products of sunlight scattering in the atmosphere, such as a coherent polarization pattern and chromatic gradient across the sky (Fig. 1A). Therefore, these neurons are suitable for heading-direction coding relative to the sun and considered a basis of orientation behavior dependent on a sky compass (Heinze, 2017; Honkanen et al., 2019).

In desert locusts, different celestial cues complement each other for robust head-direction coding. The locust CX contains a topographic arrangement of neurons tuned to the azimuth of bright light spots, simulating direct sunlight, across the vertical slices of the PB (Pegel et al., 2019). The neurons are also tuned to the angle of polarization (AoP) of light across the entire hemisphere above the animal (Bech et al., 2014; Zittrell et al., 2020). The AoP tunings of individual neurons are coherently arranged across the dorsal hemisphere and match the sky polarization pattern produced by a particular position of the sun (Bech et al., 2014; Zittrell et al., 2020). This polarization-based solar azimuth is topographically arranged consistent with the direct sunlight compass (Zittrell et al., 2020).

The polarization-vision pathway is largely conserved across insects (el Jundi et al., 2014; Hardcastle et al., 2021; Homberg et al., 2011). It originates from the dorsal rim area of the compound eye, where specialized, homochromatic photoreceptors detect polarized light, and runs through the optic lobe, anterior optic tubercle and bulb to finally enter the CBL via tangential neurons (TL neurons, corresponding to R neurons in *Drosophila*). Tuning of TL neurons to the AoP in the zenith above the animal and to the azimuth of light spots shows a 90 deg angular difference (Pegel et al., 2018) corresponding to the natural relationship between the zenithal AoP and the solar azimuth in the sky (Fig. 1A). To elucidate whether the matching AoP and direct sunlight signaling in postsynaptic CX compass neurons studied by Zittrell et al. (2020) is inherited from TL neurons, or emerges through integration of TL inputs to postsynaptic CX compass neurons, we studied the receptive field structures of TL neurons by applying light stimuli simulating polarization and direct sunlight across the sky.

## MATERIALS AND METHODS

### Animals and preparation

Adult male and female desert locusts (*Schistocerca gregaria* Forsskål 1775) were reared under crowded conditions at 28°C in a 12 h light:12 h dark cycle. After removing legs and wings, animals were mounted on a metal holder with dental wax. The head capsule was opened frontally; ocelli and antennae were removed. Fat, tracheal tissues and muscles were partially removed to expose the brain. We also removed the esophagus and gut through the abdomen to reduce peristaltic movements. A twisted metal wire was placed under the brain to stabilize it. A small part of the neural sheath was removed with fine tweezers to allow brain penetration by the recording electrode. During dissection and intracellular recording, the brain was immersed in locust saline (Clements and May, 1974).

### Intracellular recording

Sharp glass microelectrodes were drawn from borosilicate capillaries (Hilgenberg, Malsfeld, Germany) by a Flaming/Brown horizontal puller (P-97, Sutter Instruments, Novato, CA, USA). We filled electrode tips with 4% Neurobiotin (Vector Laboratories, Burlingame, CA, USA) in 1 mol l^−1^ KCl and shanks with 1 mol l^−1^ KCl. Neural signals were amplified (×10) and filtered (20 kHz low-pass) by an amplifier (SEC 05 L, NPI Electronic, Tamm, Germany). The signals were digitized at 20 kHz and stored on a PC by an A/D converter and associated software (Power1401-mkII and Spike2 version 7.06, Cambridge Electronic Design, Cambridge, UK).

### Visual stimulation

We used three types of light for visual stimulation: linearly polarized blue light, unpolarized green light, and unpolarized blue light. They were switchable during recording. Polarized blue light was used to test AoP sensitivity of single cells. Blue LED light (ELJ-465-627, Roithner LaserTechnik, Vienna, Austria) was passed through a diffuser and a polarizer (HN38S, Polaroid, Cambridge, MA, USA). The light covered a visual angle of 5.2 deg, and the light intensity was 8.4×10^13^ photons cm^−2^ s^−1^ with a peak at 461 nm. A single AoP stimulus was a full rotation of the polarizer at an angular velocity of 40 deg s^−1^ clockwise or counterclockwise. We started rotating the polarizer several seconds after the light was turned on to exclude phasic responses to lights on. The initial orientation of the polarizer was always 0 deg, which is parallel to the animal's anterior–posterior body axis when the light was positioned at the animal's zenith. An unpolarized green light was used to test sensitivity to direct sunlight, reported previously in TL neurons by Pegel et al. (2018, 2019). An unpolarized blue light was used for comparison. The light sources were green and blue LEDs (green: Nichia NCSE119A, Lumitronix, Hechingen, Germany; blue: OSLON SSL 80 LD CQ7P, OSRAM Opto Semiconductors, Regensburg, Germany). Both lights covered a visual angle of 1.05 deg. The green light intensity was 1.7×10^14^ photons cm^−2^ s^−1^ with a peak at 518 nm, and the blue one was 1.2×10^15^ photons cm^−2^ s^−1^ with a peak at 440 nm.

The stimulus setup was modified from that described by Bech et al. (2014) and Zittrell et al. (2020). The animal was positioned in the center of the setup with its anterior–posterior body axis oriented vertically (Fig. 1B). A stimulation device containing the three lights was mounted on a perimeter apparatus. After stimulating the animal from the zenith, we shifted the light device in left–right directions along the perimeter and tilted the whole perimeter in anterior–posterior directions (arrows in Fig. 1B). This allowed stimulation of the animal from various positions in its dorsal visual field.

### Histology

We injected Neurobiotin into the recorded cell by applying a positive current of up to 1 nA for 0.5–4 min. The brains were dissected out and submerged overnight at 4°C in fixative containing 4% paraformaldehyde (PFA), 0.25% glutaraldehyde, and 0.2% saturated picric acid in 0.1 mol l^−1^ phosphate buffered saline (PBS, 0.15 mol l^−1^ NaCl in 0.1 mol l^−1^ sodium phosphate buffer, pH 7.4). Optionally, the fixed brains were stored at 4°C in sodium phosphate buffer until further processing. After the fixation, the brains were rinsed in PBS and then incubated in Cy3-conjugated streptavidin (Dianova, Hamburg, Germany; 1:1000 in PBS with 0.3% Triton X-100) for 3 days at 4°C. The incubated brains were rinsed in PBS with 0.3% Triton X-100 followed by PBS, dehydrated in an ascending ethanol series (30%, 50%, 70%, 90%, 95%, 2×100%; 15 min each), and cleared in a 1:1 mixture of 100% ethanol and methyl salicylate for 20 min, followed by pure methyl salicylate for 35 min. Finally, we embedded the brains between two coverslips in Permount (Thermo Fisher Scientific, Waltham, MA, USA).

For double labeling of the recorded cells combined with GABA immunostaining, the staining method was modified from a previous study (Takahashi et al., 2017). Neurobiotin-injected brains were submerged overnight at 4°C in 4% PFA in 0.1 mol l^−1^ sodium phosphate buffer (pH 7.4). Immediately after fixation, the brains were rinsed in PBS, embedded in albumin-gelatin (4.8% gelatin and 12% ovalbumin in demineralized water) and fixed overnight at 4°C in 8% formaldehyde diluted in 0.1 mol l^−1^ sodium phosphate buffer. The brains in the gelatin block were cut into 80–130 µm sections by a vibrating-blade microtome (VT 1000S, Leica Microsystems, Wetzlar, Germany). Brain sections were rinsed in PBS with 1% Triton X-100 (PBST) and then blocked for 1 h at room temperature in 2% normal goat serum diluted in PBST with 0.25% bovine serum albumin (PBST-BSA). Afterward, the sections were incubated for 5 days at 4°C in a mixture of anti-synapsin monoclonal antibody (RRID: AB_2315425; provided by Erich Buchner and Christian Wegener, University of Würzburg, Germany; 1:50) and anti-GABA polyclonal antibody generated in rabbit (A2052, RRID: AB_477652, Sigma, Steinheim, Germany; 1:1000) diluted in PBST-BSA. Following the incubation, the sections were rinsed in PBST-BSA and incubated for 5 days at 4°C in a mixture of goat-anti-mouse-Cy5 (Dianova, 1:300), goat-anti-rabbit-Cy2 (Dianova, 1:300), and streptavidin-Cy3 (1:1000) in PBST-BSA. After incubation, the sections were rinsed, dehydrated, cleared, and embedded in Permount.

### Image acquisition and processing

We scanned preparations with a confocal laser scanning microscope (TCS SP5, Leica Microsystems). Cy3 signals were detected with a diode-pumped solid-state laser (561 nm). In GABA- and synapsin-labeled sections, Cy2 and Cy5 signals were detected with an argon laser (458 nm) and a helium-neon laser (633 nm), respectively. Spatial resolution (pixel size) in the *x*, *y*-plane was about 0.51 µm×0.51 µm for the morphology of whole neurons and approximately 0.13 µm×0.13 µm for magnified cell bodies. Step size was 1.5–3.0 µm along the *z*-axis. Scanned images were stacked two-dimensionally in an image-processing software (ImageJ v. 1.52a, NIH, Bethesda, MD, USA; Schneider et al., 2012). Input levels of the image stacks were uniformly adjusted in photo-editing software (GNU Image Manipulation Program version 2.10.22, GIMP Development Team). We deduced innervation layers of neurons from the position of their arborizations within neuropils, identifiable through tissue autofluorescence or visualized synapsin.

### Data pre-processing

Physiological data were analyzed when the recorded neuron was successfully labeled. More than one neuron was stained in some preparations, probably because of leakage of Neurobiotin into neighboring cells. We included these cases in the analyses if we identified the recorded neuron based on Neurobiotin (Cy3) signal intensity or if all stained cells belonged to the same cell type and had cell bodies in the same brain hemisphere.

For pre-processing of recorded data, action potentials were detected in Spike2 by a threshold-based feature detection script (FeatureDetect.s2s, downloaded from the CED website). Detection quality was verified by visual inspection. We performed all subsequent analyses in MATLAB v. 2021b (The MathWorks, Natick, MA, USA) and R v. 4.1.1 (https://www.r-project.org/). The significance level for statistical tests was α=0.05.

### Data plots

Data were plotted as boxplots in the following way. Boxes range from the 25th (Q1) to 75th (Q3) percentile of the data. Horizontal lines in the boxes indicate the median. Whiskers extend to the adjacent value that is the most extreme data point, which is not less than Q3−1.5×(Q3−Q1) and greater than Q3+1.5×(Q3−Q1). Numerals of *x*-axis labels represent sample numbers.

Spherical coordinates of the dorsal visual field (Fig. 1B,C) were transformed on a polar-coordinate grid to show the data on a flattened hemisphere from above (Fig. 1D) following Zittrell et al. (2020). The center (pole) of the grid corresponds to the zenith (elevation=90 deg). The radius from the pole (ρ) is defined as 1 – elevation/90 deg (0≤ρ≤1) and the angle (θ) equals the spherical azimuth (0≤θ<360 deg). Elevation and azimuth are indicated relative to the animal's head.

### Response to AoP: sensitivity

Spike times during stimulation were transformed into the orientation of the polarizer (spike angles) based on angular velocity (40 deg s^−1^) and direction (clockwise or counterclockwise) of polarizer rotation. Spike angles were used to calculate spike rates per 10 deg bin (36 bins from 0 to 360 deg). For spike rate calculations, we pooled spike activities to equal numbers of clockwise and counterclockwise rotations to avoid spike angle shifts due to rotation direction.

To judge neural responses to the AoP orientation, we calculated the square of the circular-linear correlation coefficient (0≤*r*_cl_^2^≤1) between bin center angles and spike rates per bin using the function ‘circ_corrcl’ in the Circular Statistics Toolbox of MATLAB (Berens, 2009) (see Supplementary Materials and Methods). Bin center angles were doubled for the calculation because the AoP is axial data: 0 deg=180 deg (Batschelet, 1981). Spike activities were considered an AoP response when the *P*-value of *r*_cl_^2^<0.05. Because the *P*-value of *r*_cl_^2^ depends on the sample number used for the calculation (Berens, 2009), and we always used spike rates of 36 bins, *P*<0.05 is equivalent to *r*_cl_^2^>0.1664.

### Response to AoP: tuning properties

To yield a tuning curve of AoP responses, we fitted von Mises distributions to spike angle data. The von Mises distribution is known as a circular normal distribution, commonly used for circular data analysis. In a von Mises distribution, the probability of angles θ depends on two parameters: peak position μ and concentration κ. Increasing values of κ represent increased probability of angles around μ, whereas *κ*=0 results in the uniform distribution.

To describe our bimodal AoP response data, we mixed two von Mises distributions as described in Fitak and Johnsen (2017) and Schnute and Groot (1992):
(1)

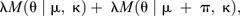
where *M*(θ | μ, κ) denotes a von Mises distribution with peak μ and concentration κ. The mixed von Mises distribution represented by Eqn 1 possesses bimodal peaks of the same height (λ) and width (κ) in symmetric positions (μ and μ+π). The best-fit parameters λ, μ and κ for a tuning curve of the data were found by the maximum likelihood method; the likelihood was calculated by the function ‘circ_mle’ in the CircMLE package of R (Fitak and Johnsen, 2017). The best-fit peak position μ (0≤μ<180 deg) was termed Φ_max_ (preferred angle) and Φ_min_ (anti-preferred angle) was defined as 90 deg distant from Φ_max_. The half width at the half amplitude of the peak was used as tuning width. Tuning width was measured with increments of 1 deg to simplify the calculation.

Further, we quantified excitatory and inhibitory modulations of spike rate caused by AoP presentation based on the tuning curve and the background activity (BA). The BA level for this analysis was the averaged firing rate (spikes s^−1^) calculated from one or several 1 s bins before the polarized light was turned on. We defined Φ_max/min_ activity as the difference between the spike rate at Φ_max/min_ and the BA. These values indicate excitatory (upward arrow) or inhibitory (downward arrow) modulation from the BA. In most cases, Φ_max_ and Φ_min_ activities were excitatory and inhibitory, respectively. However, both Φ_max_ and Φ_min_ activities can be excitatory or inhibitory, depending on the tuning curve position relative to the BA. To compare modulation strengths and direction between individuals, we scaled Φ_max_ activities by the amplitudes of the individual tuning curves (Φ_max_ activity/amplitude). The amplitude of a tuning curve is defined as the difference of Φ_max_ and Φ_min_ activities. Here, we did not use the BA for scaling because some individuals had no background spiking before the stimulus.

### Response to AoP: receptive fields

To visualize neural receptive fields to AoP, we plotted surface heatmaps based on AoP sensitivity (*r*_cl_^2^). The *r*_cl_^2^ values of tested positions were linearly interpolated in between on the flattened hemispheres (Fig. 1D) using the function ‘scatteredInterpolant’ in MATLAB.

Further, we determined highly AoP sensitive parts of the receptive fields in each cell for comparison with downstream CX neurons analyzed in Zittrell et al. (2020), in which AoP receptive fields were defined as the regions with 75% or more *r*_cl_^2^ value to the response maxima of individual recordings. To find the corresponding regions, we followed the procedures of Zittrell et al. (2020). Normalized *r*_cl_^2^ values of tested positions were linearly interpolated to the points distributed over the surface of the dorsal visual field, and boundary lines were drawn to enclose all points with normalized *r*_cl_^2^≥0.75.

A detailed description is provided by Zittrell et al. (2020). Briefly, first, we distributed 2500 points evenly over the surface of a spherical dorsal visual field hemisphere using a hemispherical Fibonacci grid proposed by Swinbank and Purser (2006). The spherical coordinates for the 2500 points were transformed to two-dimensional coordinates on a flattened hemisphere (Fig. 1D) to simplify the following calculations. Out of the 2500 points, we only used the points inside the convex hull of the positions tested in individual recordings; the convex hull was built by the Delaunay triangulation method in the same way as surface heatmaps.

Next, the *r*_cl_^2^ values at the tested positions were normalized to the response maxima of individual recordings and linearly interpolated over the generated points using the function ‘scatteredInterpolant’ in MATLAB. We picked up the points with normalized *r*_cl_^2^≥0.75, which resulted in one or more clouds of data points. To categorize those clouds into individual fields, we used an agglomerative hierarchical clustering approach. This clustering method successfully merges pairs of the closest or most similar data point sets (clusters) into single clusters. In the first step, each data point was considered its own cluster. The distances or similarities between pairs of clusters are defined by a linkage criterion. In the current study, the linkage criterion ‘minimum distance (single-linkage) method’ was applied to all recordings, defining the clusters' distance as the minimum distance between a data point in one cluster and a data point in the other cluster. The great-circle distance was adopted as a distance measure, and the original spherical coordinates of data points were used to calculate the distances here (see Supplementary Materials and Methods). The clusters were split (not merged) when their distance was greater than the visual angle covered by the polarized light of the stimulus setup (5.2 deg×π/180). Finally, we drew a boundary line to enclose all data points of each cluster to mark the higher AoP sensitivity regions corresponding to the study by Zittrell et al. (2020).

### Response to AoP: best-matching polarization patterns

Many neurons of the locust CX show coherent arrangements of preferred AoP orientations across the dorsal visual field (Bech et al., 2014; Zittrell et al., 2020). These tuning arrangements act as filters that match the sky polarization pattern generated by particular solar coordinates relative to the animal (Bech et al., 2014; Zittrell et al., 2020). We defined the best-matching polarization pattern as the sky polarization pattern that would evoke the highest neuronal activity in a cell. We also defined the preferred sun encoded by AoP responses as the solar coordinates that generate the best-matching polarization pattern.

To find the preferred sun encoded by AoP responses, we used the procedure described in Zittrell et al. (2020), which was adapted from the original of Bech et al. (2014). This procedure calculates deviations between a neural response pattern (Φ_max_ angles) and various model sky polarization patterns. The model pattern with the minimum deviation from the neural responses is considered the best-matching pattern, and the corresponding solar coordinates are the position of the preferred sun.

A detailed description of the procedures is provided in Zittrell et al. (2020). Briefly, first, we generated sky polarization patterns (angles and degrees of polarization) based on the single-scattering Rayleigh model (Strutt, 1871) (Fig. 1A and see Supplementary Materials and Methods). We prepared 32,760 model patterns from equally spaced solar positions (azimuth 360 ways×elevation 91 ways). Next, for each model pattern, we calculated the absolute angular differences (from 0 deg to 90 deg) between the Φ_max_ angles of AoP responses (*r*_cl_^2^>0.1664) and the model angles of polarization. Finally, we averaged the absolute angular differences to yield the deviation of the model pattern.

Before the averaging process, the absolute angular differences were weighted in each position; weighting factors were (1) response *r*_cl_^2^ value, (2) model degree of polarization, and (3) the normalized sum of the great-circle distances to the nearest 22% of tested positions. The third weighting factor was introduced to counterbalance the overrepresentation of values from densely sampled areas. The value of 22% was chosen in accordance with the original procedure (Bech et al., 2014), where every data set contained AoP responses from 37 positions, and the nearest eight positions were used to calculate the weighting factor. In the current study and the study of Zittrell et al. (2020), the total number of tested positions varied due to the instability of intracellular recordings. Hence, we chose 8/37≈22% of the total number of tested positions of each data set as the number of nearest positions to calculate the weighting factor.

### Response to AoP: evaluation of pattern matching results

Neural AoP responses can encode unambiguous solar coordinates when the minimum deviation between the response pattern and the best-matching polarization pattern is small enough. To evaluate the minimum deviation and, thus, the matched-filter quality of a neuron, Zittrell et al. (2020) performed a bootstrap procedure. In this procedure, the *P*-value of the minimum deviation of the best matching pattern is calculated as the probability that a lower value is observed in a population of the minimum deviations of randomized response patterns. When *P*<0.05, the neuron is considered as a reliable matched filter encoding unambiguous solar coordinates based on the sky polarization pattern.

A detailed description of the procedures is provided in Zittrell et al. (2020). Briefly, first, we generated 5000 randomized response patterns from an actual neural response pattern; a randomized pattern was generated by randomly drawing (with replacement) AoP responses from the pool of the actual neural responses (*r*_cl_^2^>0.1664) and distributing them on all neural response positions. Next, for each randomized response pattern, we calculated the best-matching polarization pattern and its deviation in the same way as described in the previous section, yielding a bootstrap population of the minimum deviations. Finally, the *P*-value of the minimum deviation for the actual neural response pattern was calculated as follows:
(2)

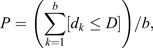
where *D* is the minimum deviation of the actual data, *k* is the bootstrap sample index, *b* is the number of randomized samples (5000) and *d_k_* is the minimum deviation of the *k*th sample. Recordings were excluded from the analysis if there were only one or two AoP responses because the number of possible randomized sample variations was too small (≤2^2^).

In this procedure, the *P*-value tends to be large when the preferred sun encoded by the actual AoP responses is near the elevation of 0 deg in anterior (azimuth 0 deg), left (90 deg), posterior (180 deg) and right (270 deg) directions from the animal (Fig. 1D). That is because all Φ_max_ angles are approximately 0 deg or 90 deg in these cases, resulting in a small difference between the actual neural response pattern and the randomized patterns (e.g. TL2a_18 in Fig. S3). However, for comparison to the results of downstream CX neurons analyzed in Zittrell et al. (2020), we did not add any modification to the procedure.

### Response to stationary light spots

To analyze neural responses caused by light spots, we counted spikes during 1 s intervals before and after the light was turned on (control and post-ON) and estimated a 68% confidence interval (CI) of the mean of post-ON spike counts. A 68% CI equivalents to mean±s.d. of normal distribution data. Spike activities during a post-ON interval were considered an inhibitory response when the CI was on the left to the mean of control spike counts, while they were considered an excitatory response when the CI was on the right to the control mean. When the CI contained the control mean, spike activities were considered no response.

We estimated a 68% CI of spike counts by the χ^2^ distribution method (Sahai and Khurshid, 1993), because the simple mean±s.d. method is inappropriate to estimate a CI of count data when the mean value is small. The χ^2^ distribution method estimates lower and upper 68% confidence limits of mean λ separately as follows:
(3)

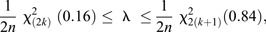
where χ^2^_(*k*)_ (α) denotes the 100α percentile of the χ^2^ distribution with *k* degrees of freedom, *n* is the sample number and *k* is the sample sum (Sahai and Khurshid, 1993).

To visualize neural receptive fields to light spots, we plotted surface heatmaps based on spike count modulation (Δspikes s^−1^) in the same way as the AoP receptive fields. Spike count modulation was defined as the difference from the mean of control spike counts to the nearest 68% confidence limit of the mean of post-ON spike counts: the upper limit in inhibitory responses and the lower limit in excitatory responses. Spike count modulation of no response was defined as 0 independent of the control and post-ON spike count means.

## RESULTS

### Morphology of AoP-sensitive TL neurons

**Fig. 2.**
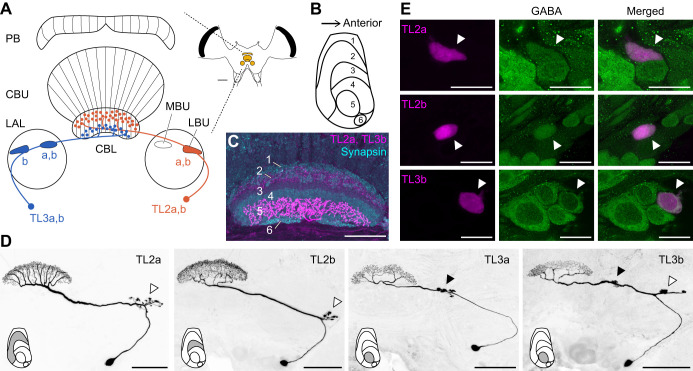
**Layers in the lower division of the central body (CBL) and tangential (TL) neuron subtypes.** (A) Schematic drawing of the locust central complex (CX) and TL neurons investigated in this study, modified from Pegel et al. (2019). Inset shows a frontal diagram of the locust brain with the CX and its associated bilateral structures (lateral complex) in yellow. Dots in CBL represent varicose arborizations and are thus likely presynaptic output regions. The filled lateral and medial bulbs (LBU, MBU) indicate input synapses arranged in microglomerular complexes. (B,C) CBL layers numbered 1–6. Sagittal diagram (B) and frontal confocal image stack (C), in which TL2a and TL3b neurons (layers 2 and 5) were stained by Cy3-labeled streptavidin–Neurobiotin. (D) Confocal image stacks of all TL neuron types investigated in this study: TL2a, TL2b, TL3a, and TL3b neurons with soma in the left hemisphere. Insets show sagittal views of the innervated CBL layer. Arrowheads point to input synapses in the lateral (white) and medial (black) bulbs. (E) GABA immunoreactivity of TL neuron subtypes. Arrowheads point to cell bodies of double-labeled neurons. Scale bars: 400 µm (inset of A), 50 µm (C), 100 µm (D) and 20 µm (E). CBU, upper division of the central body; LAL, lateral accessory lobe; PB, protocerebral bridge.

We recorded intracellularly from 59 tangential neurons of the CBL in the locust CX (Fig. 2A). All neurons were sensitive to the AoP. First, we determined their cell types. The locust CBL consists of six horizontal layers (Fig. 2B,C) (Müller et al., 1997) and six types of tangential neurons termed TL1–TL5 (Müller et al., 1997; von Hadeln et al., 2020) and TL7 (Hensgen et al., 2021) have been distinguished based on the location of their input arborizations and cell bodies. All of our recordings were from TL2 and TL3 neurons (*N*=38 and 21, respectively).

Each of TL2 and TL3 populations was estimated to consist of up to 40 individuals per brain hemisphere (Homberg et al., 1999). TL2 neurons are defined by their cell body along the ventro-medial face of the lateral complex and ramifications in small areas of the lateral bulb (Müller et al., 1997; von Hadeln et al., 2020). Two subtypes of TL2 neurons have been distinguished: TL2a and TL2b (Pegel et al., 2019). TL2a neurons arborize in dorsal parts of the lateral bulb and invade layer 2 of the CBL, while TL2b neurons arborize in ventral parts of the lateral bulb and invade layer 3 (orange neuron in Fig. 2A). Based on these criteria, we recorded from 34 TL2a neurons and four TL2b neurons (Fig. 2D).

TL3 neurons share a common cell body position with TL2 neurons, but their dendrites ramify in the medial bulb (Müller et al., 1997; von Hadeln et al., 2020). Three subtypes of TL3 neurons have been distinguished, termed TL3a, TL3b (von Hadeln et al., 2020) and TL3c (Hensgen et al., 2021). We recorded from four TL3a and 17 TL3b neurons but no TL3c neurons (e.g. Fig. 2D). TL3a neurons exclusively ramify in the medial bulb, while TL3b neurons have additional ramifications in the lateral bulb or along the isthmus tract (blue neuron in Fig. 2A). All TL3a and TL3b neurons recorded in this study innervated layer 5. Pegel et al. (2019) reported three AoP-sensitive TL3 neurons innervating layers 4 and 5 of the CBL. We reanalyzed those neurons and concluded that their innervation was confined to layer 5 as in all TL3 neurons studied here. We found symmetric and asymmetric branching patterns (von Hadeln et al., 2020) in both TL3a and TL3b neurons.

Based on morphological criteria, TL2 neurons likely correspond to R2 cells and TL3 neurons to R3 cells in *Drosophila* (Omoto et al., 2017). R2 and R3 cells are thought to be GABAergic (Hanesch et al., 1989). Homberg et al. (1999) reported GABA immunoreactivity of single TL2 and TL3 neurons but did not distinguish between the different subtypes. Therefore, we tested GABA immunoreactivity of 22 TL neurons (Fig. 2E). We found that the cell bodies of all examined TL2 and TL3 neurons were GABA-immunoreactive: TL2a [*N*=10 (recorded) and *N*=6 (staining only)], TL2b (*N*=1 and *N*=2) and TL3b (*N*=2 and *N*=1) neurons. Unfortunately, we could not perform double labeling of TL3a neurons, but our data support similar polarization-sensitive input architectures to the ring attractor networks in the locust and the fly.

### Response to zenithal AoP

**Fig. 3.**
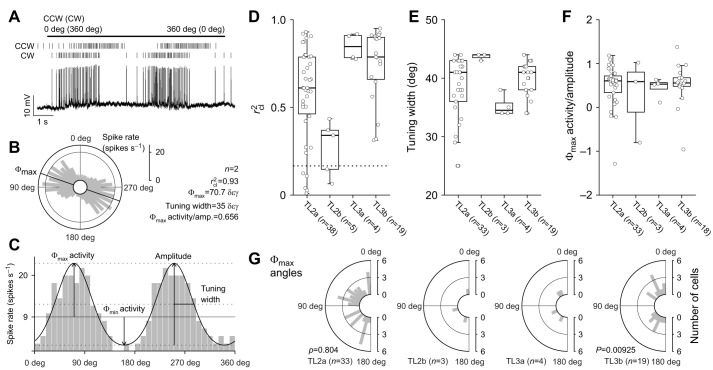
**Response to zenithal angle of polarization (AoP).** (A) Spike activities of a TL2a neuron (TL2a_19) during a 360 deg clockwise (CW) and counterclockwise (CCW) rotation of the polarizer. Spike rasters are aligned to the time of rotation represented above. (B,C) Spike trains represented in A shown as spike rate histograms with 10 deg bins presented as polar and linear plots, respectively. The response showed a strong correlation with the orientation of the polarizer (the square of circular–linear correlation coefficient *r*_cl_^2^=0.93, *P*=5.37×10^−8^). The best-fit tuning curve (mixed von Mises distributions) and the background activity (BA) level before the stimulus (horizontal line) are superimposed on the histogram in C. (D–G) Tuning properties of AoP responses. (D) The square of circular-linear correlation coefficient (0≤*r*_cl_^2^≤1) between spike rate and polarizer orientation as AoP sensitivity. Values of *r*_cl_^2^>0.1664 (horizontal dotted line), equivalent to *P*<0.05, are considered as AoP responses. Individuals with *r*_cl_^2^≤0.1664 (*N*=7) are excluded from other plots. (E) Tuning width (deg) of the tuning curve. (F) Φ_max_ activity/amplitude of the tuning curve. The amplitude of the tuning curve (right arrow in C) indicates the difference of Φ_max_ and Φ_min_ activities. Φ_max_ and Φ_min_ activities (left and center arrows in C) indicate spike rate modulation from the BA (horizontal line in C); positive and negative values are excitatory and inhibitory modulation, respectively. Values of Φ_max_ activity/amplitude range from 0 to 1 when the spike rate at Φ_max_ is higher than the BA and the spike rate at Φ_min_ lower than the BA. When the spike rates at Φ_max_ and Φ_min_ are lower than the BA, the plot value is <0, and when the spike rates at Φ_max_ and Φ_min_ are higher than the BA, the plot value is >1. In one TL3 neuron, the BA before the stimulus was not available because of recording noise, and thus it was excluded from the analysis. (G) Peak position of the tuning curve (0 deg≤Φ_max_<180 deg) shown in histograms with 10 deg bins. The *P*-values of Rayleigh test of uniformity are given for the Φ_max_ distributions of TL2a and TL3b neurons.

TL neuron data presented previously (Pegel et al., 2018, 2019) were included in our analysis hereafter: seven recordings in which the polarizer was rotated at the same angular velocity (40 deg s^−1^) as in our stimulus setup. First, we investigated neural sensitivity and tuning properties to zenithal AoP (Fig. 3A) tested at the beginning of the recordings (∼100 s) to exclude effects of stimulus position and fluctuations of background activity (BA) (Supplementary Materials and Methods, Fig. S1). To judge neural responses to the AoP orientation, we used the square of circular–linear correlation coefficient (*r*_cl_^2^) between spike rate and the polarizer orientation (Fig. 3B,C). Out of 66 neurons, five TL2a and two TL2b neurons were not sensitive to the zenithal AoP (Fig. 3D, *r*_cl_^2^<0.1664), although they responded to the AoP at other stimulus positions. All TL3a and TL3b neurons were sensitive to the zenithal AoP. In the TL2a population, individual *r*_cl_^2^ values were widely distributed, ranging from 0.00935 to 0.930 with the median 0.611, while the TL2b population showed lower AoP sensitivity (median=0.341) than the other cell types. The *r*_cl_^2^ values of TL3a and TL3b neurons were higher (medians=0.847 and 0.7888, respectively) than those of TL2 neurons (Fig. 3D).

We analyzed tuning properties of AoP responses by fitting mixed von Mises distributions to spike activities (Fig. 3C). Tuning width (horizontal line segment in Fig. 3C) was largest in TL2b neurons and smallest in TL3a neurons (Fig. 3E). TL2a and TL3b neurons shared similar intermediate tuning widths (Fig. 3E).

Φ_max (min)_ activity (left and center arrows in Fig. 3C) indicates spike rate modulations from the BA (horizontal line in Fig. 3C) at Φ_max (min)_. Positive (upward arrow) and negative (downward arrow) Φ_max/min_ activities are excitatory and inhibitory modulations, respectively. Fig. 3F shows Φ_max_ activities scaled by the amplitudes of individual tuning curves (Φ_max_ activity/amplitude); here, the amplitude of a tuning curve is the difference of Φ_max_ and Φ_min_ activities (right arrow in Fig. 3C). Φ_max_ activity/amplitude between 0 and 1 means excitatory modulation at Φ_max_ and inhibitory modulation at Φ_min_. When the tuning curve is below the BA, inhibitory modulations occur both at Φ_max_ and Φ_min_, resulting in Φ_max_ activity/amplitude values <0. When the tuning curve is above the BA, the neuron was excited both at Φ_max_ and Φ_min_, resulting in values >1 because, in this case, Φ_max_ activity is larger than the amplitude of the tuning curve. In our data set, Φ_max_ activity/amplitude of most individuals was between 0 and 1 independent of cell type, which means that the zenithal AoP usually induced excitation at Φ_max_ and inhibition at Φ_min_ in AoP-sensitive TL neurons.

Finally, Fig. 3G shows Φ_max_ histograms for each cell type. The Φ_max_ distribution of TL2a neurons was uniform (Rayleigh test of uniformity to doubled Φ_max_, *Z*=0.0804, *P*=0.784), while that of TL3b neurons had a gap around 90 deg (*Z*=0.487, *P*=0.00925). We did not test the distribution of TL2b and TL3a neurons owing to the small sample size. In summary, TL2a neurons cover the full range of zenithal AoP orientations (from 0 deg to 180 deg) with various sensitivity levels (*r*_cl_^2^). On the other hand, TL3 neurons have higher AoP sensitivity, but their activities do not code zenithal AoP orientation around 90 deg.

### Receptive fields of AoP responses

**Fig. 4.**
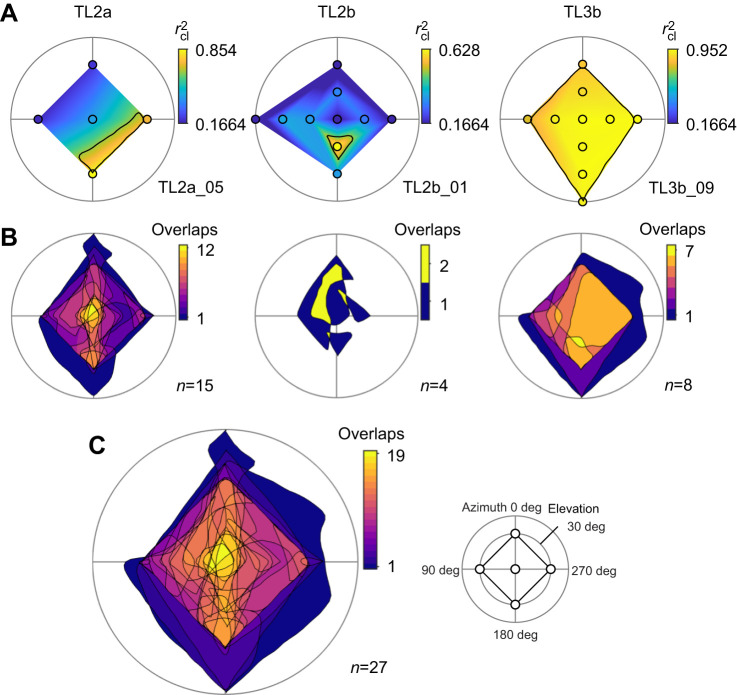
**Receptive fields for the AoP.** (A) Polarization sensitivity maps of three recordings (top view on flattened hemispheres; see inset in C and Fig. 1D for clarification of the coordinate system). The sensitivity to polarization angle (*r*_cl_^2^ value) is color-coded at each tested position (circles) and linearly interpolated in between. The fields with *r*_cl_^2^≤0.1664 (dark blue) are considered no AoP response parts. Black lines are smoothed 75% isolines of the normalized *r*_cl_^2^ values. (B,C) Superimposed boundary lines of 75% fields of each cell type in B and all analyzed neurons in C with a color-coded degree of overlap. *n* indicates number of neurons. Inset in C shows the minimum requirement of stimulus positions (circles) for the receptive field analysis: the anterior, left, posterior and right direction at an elevation of 30 deg relative to the animal's head as well as the zenith (elevation 90 deg). See Figs S2–S4 for sensitivity maps of all individuals.

Next, we investigated the receptive fields of the neurons to the AoP (Fig. 4). Following zenithal AoP stimulation, we stimulated from various positions within the dorsal visual hemisphere of the animal, which yielded an AoP sensitivity (*r*_cl_^2^) map for each neuron (examples in Fig. 4A). Recordings from 27 individual neurons were used for the analysis in which the AoP sensitivity was measured at least at five positions: at the zenith and at elevations of 30 deg in anterior (azimuth 0 deg), left (90 deg), posterior (180 deg), and right (270 deg) directions from the animal (inset in Fig. 4C). No TL3a neuron was available for this analysis.

To compare the receptive field organizations for AoP sensitivity of TL neurons with those in downstream neurons of the CX (Zittrell et al., 2020), we defined the receptive fields as those areas that had *r*_cl_^2^ values of at least 75% of the response maxima of each cell (inside black boundary line in Fig. 4A; see Figs S2–S4 for all 27 individuals) as done by Zittrell et al. (2020). Similar to the results of the downstream CX neurons, the receptive fields for AoP sensitivity generally varied in size, shape and position in individuals, but with some cell type-specific trends. In TL2a/2b neurons, the *r*_cl_^2^ values tended to be highly affected by stimulus position, resulting in relatively small susceptible parts (Fig. 4A,B, Figs S2 and S3). In contrast, the *r*_cl_^2^ values of most TL3b neurons were high across the dorsal visual field, resulting in larger sizes of receptive fields (Fig. 4A,B, Fig. S4).

We superimposed the boundaries of the 75% *r*_cl_^2^ fields of all 27 neurons (Fig. 4C) in the same way as done by Zittrell et al. (2020). Similar to the downstream CX neurons, the overlap of the receptive fields was nearly bilaterally symmetrical to the midline and maximal around the zenith (Fig. 4C), suggesting that the AoP receptive field structures are conveyed from TL populations to the downstream CX network.

### Matched-filter properties of AoP sensitivity

**Fig. 5.**
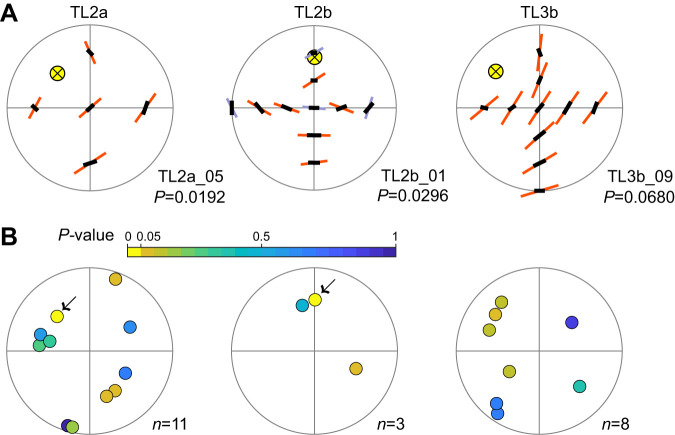
**AoP response patterns and best-matching polarization patterns.** (A) Pattern fitting results of three individual neurons (top view on flattened hemispheres). The same individuals are shown as those in Fig. 4A. The Φ_max_ of the tuning curve is shown by orange (*r*_cl_^2^>0.1664) or gray (*r*_cl_^2^≤0.1664) bar orientation at each tested position. The best-matching sky polarization patterns (black bars) are superimposed on the responses. The bar length was scaled by *r*_cl_^2^ value (response pattern) and degree of polarization (polarization pattern). A crossed yellow circle indicates the solar position used to generate the polarization pattern. The *P*-values are the results of the bootstrap test of the minimum deviation. (B) The solar positions with the minimum deviations of single cells. Data points are color-coded by *P-*value of the minimum deviations with increments of 0.05; yellow data points of *P<*0.05 are indicated by arrows. *n* indicates the number of neurons. See Figs S2–S4 for response patterns of all individuals.

The preferred AoP orientations of TL neurons were coherently arranged across the dorsal visual field (Fig. 5, Figs S2–S4), similar to those of CX neurons investigated in previous studies (Bech et al., 2014; Zittrell et al., 2020). In postsynaptic CX compass neurons, these tuning arrangements likely act as filters matched to the sky polarization pattern generated by particular solar coordinates. We estimated the sun positions encoded by AoP responses by calculating the best-matching sky polarization pattern with the minimum deviation from the neural response pattern (pattern matching procedure; Bech et al., 2014; Zittrell et al., 2020) and quantitatively assessed the matched filter qualities of the neurons by calculating the *P*-value of the minimum deviation using a bootstrap procedure (Zittrell et al., 2020).

Fig. 5A shows examples of the AoP response patterns measured in single neurons (orange and gray bars). On each response pattern, we superimposed the best-matching sky polarization pattern (black bars) calculated by the single-scattering Rayleigh model and its corresponding sun position (crossed yellow circle). We estimated best matching sun coordinates in 22 individuals out of 27 used in the receptive field analysis in the previous section; five recordings were excluded because of low number of AoP responses (≤2).

To assess the matched filter quality of individual neurons, we applied a bootstrap procedure that evaluates the minimum deviation of the best matching polarization pattern. Fig. 5B shows the distributions of the sun positions encoded by AoP responses for all analyzed neurons. Data points are color-coded by the *P-*value of the minimum deviation of the best-matching pattern with increments of 0.05. Only two neurons passed the criterion *P*<0.05 and thus are considered a reliable matched filter of the sky polarization patterns (yellow circles, arrows in Fig. 5B; TL2a_05: minimum pattern deviation=1.84 deg, *P*=0.0192; TL2b_01: minimum pattern deviation=7.63 deg, *P*=0.0296). Therefore, the proportion of reliable matched filter coding in TL neurons (2 out of 22 recordings) is considerably lower than in downstream CX neurons (17 of 23 neurons; Zittrell et al., 2020). This proportion was not affected (proportion: 1 out of 10 recordings) by including only recordings with at least nine tested stimulus positions, the requirements equal to those of Zittrell et al. (2020). The morphology of the two TL2 neurons exhibiting good matched-filter properties was not distinct from that of other TL2 cells.

### Receptive fields for stationary light spots

Besides sensitivity to AoP, TL2 and TL3 neurons are sensitive to the azimuth of an unpolarized green light spot rotating around the animal's head, suited to code for solar azimuth (Pegel et al., 2018, 2019). Our data, based on stationary green and blue light spots presented at different positions across the dorsal visual field, confirm these results.

**Fig. 6.**
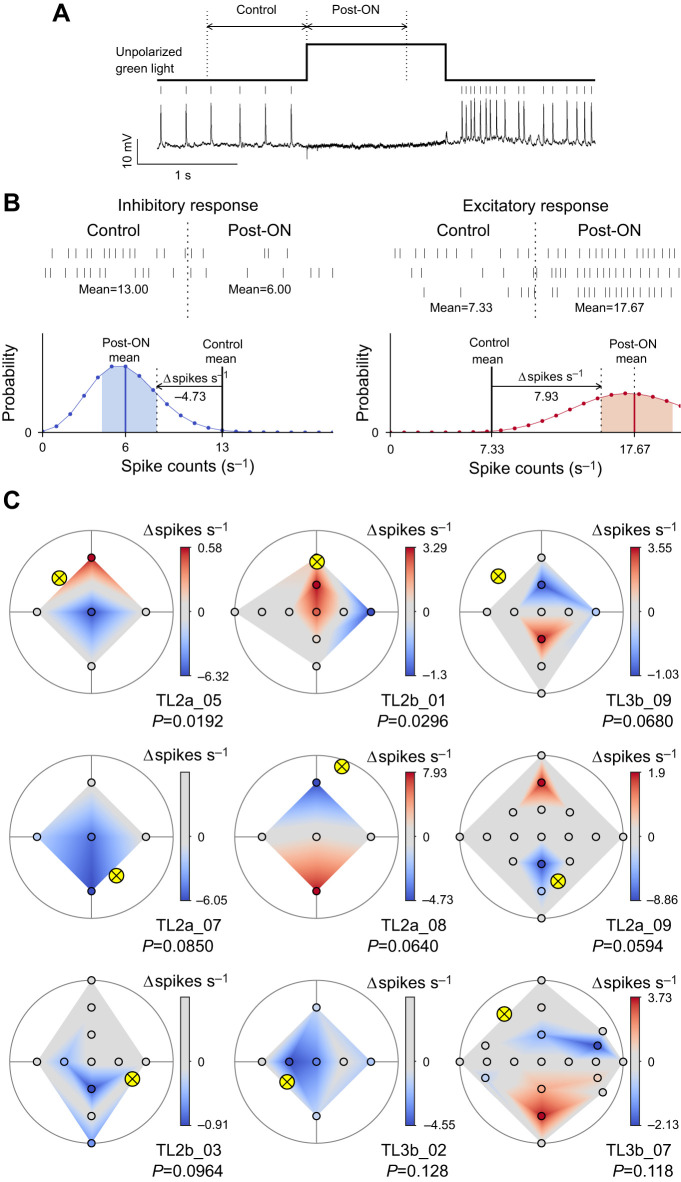
**Response to stationary light stimuli in the dorsal visual field.** (A) Spike activities of a TL2a neuron during presentation of an unpolarized green light from the zenith (square wave above activities). (B) Examples of responses to stationary light and spike count modulation. A TL2a neuron (TL2a_08 shown in C) showed an inhibitory response (left) and an excitatory response (right) to the green light spot presented from the anterior and posterior directions, respectively. The upper panels are spike rasters during 1 s control and post-ON intervals, aligned to the time of the light turned on (vertical dotted lines). A mean value of spike counts (s^−1^) is represented at the bottom of a raster series of each interval. In the lower panels, Poisson distributions of spike counts are plotted with 68% confidence interval (CI) of the post-ON mean (shaded areas). The blue CI was on the left of the mean of control spike counts and thus the post-ON spike activities were considered an inhibitory response. In contrast, the red CI was on the right of the control mean and thus the post-ON spike activities were considered an excitatory response. Spike count modulation (Δspikes s^−1^) to plot surface heatmaps in C was defined as the difference from the mean of control spike counts to the nearest 68% confidence limit of the mean of post-ON spike counts (horizontal arrows): −4.73 for the inhibitory response and 7.93 for the excitatory response in these examples. (C) Spike count modulation maps of single neurons to stationary unpolarized green light spots (top view on flattened hemispheres). The three individuals in the first row are the same as those shown in Fig. 4A and Fig. 5A. The spike count modulation (Δspikes s^−1^ value) is color-coded at each tested position (circles) and linearly interpolated in between. The preferred suns encoded by AoP responses are also shown (crossed yellow circle) with *P*-values of the minimum pattern deviation. See Figs S2–S4 for receptive fields of all individuals.

In addition, we investigated whether the preferred azimuth of the light spot corresponded to the preferred sun encoded by AoP responses in individual TL neurons. To judge neural responses to light spots, we counted spikes during 1 s intervals before and after the light was turned on (control and post-ON in Fig. 6A). When the mean of the control spike counts was outside of the 68% CI of the mean of the post-ON spike counts, post-ON spike activities were considered inhibitory or excitatory responses to a light spot presentation (Fig. 6B). Otherwise, spike activities were considered no response. To plot surface heatmaps of receptive fields, we then calculated spike count modulation values (Δspikes s^−1^) as the difference from the mean of control spike counts to the nearest 68% confidence limit of the mean of post-ON spike counts (horizontal arrows in Fig. 6B). In no response spike activities, Δspikes s^−1^ value was defined as 0 independent of the control and post-ON spike count means.

In most neurons (13 recordings), the receptive fields for stationary unpolarized green light spots comprised spatially distinct excitatory and inhibitory subfields (Fig. 6C, Figs S2–S4). Six recordings, however, showed only excitatory, five only inhibitory responses, and one TL2 neuron was completely unresponsive (Figs S2–S4). Overall, the receptive field organization for blue light spots was similar to that for green light stimuli (Figs S2–S4), but when presenting blue spots, purely inhibitory responses (6 recordings) occurred more frequently than mixed excitatory/inhibitory fields (5 recordings) and purely excitatory responses (3 recordings). TL2b neurons (2 recordings) were only excited and TL3b neurons (3 recordings) were only inhibited to blue light throughout their receptive fields.

Fig. 6C shows examples of receptive fields for unpolarized green light stimulation together with the sun positions encoded by AoP responses estimated in the previous section. These examples were chosen because their *P*-values of the minimum deviations from a particular sky polarization pattern (see in the previous section) were smaller than 0.2 (see Figs S2–S4 for all individuals). In the two TL2 neurons with a faithful polarization-matched filter quality (TL2a_05 and TL2b_01, *P*<0.05), the preferred sun encoded by AoP responses and the excitatory fields defined by green light responses were located in close proximity, suggesting integration of the two related celestial cues that match the situation in the sky. However, in three other TL2a neurons and a TL3b neuron (TL2a_07, TL2a_08, TL2a_09, and TL3b_02), the sun coordinates encoded by AoP responses were near the inhibitory fields. In the remaining cases shown in Fig. 6C, the preferred sun encoded by AoP responses was far away from the receptive fields for the green light responses. These examples illustrate striking mismatches in coding of the two celestial cues in neurons at the input stage of the CX.

Receptive fields defined by unpolarized blue light are shown in Figs S2–S4. The excitatory subfield structures differ from those of green light. As a result, the preferred sun encoded by AoP responses was far away from the excitatory fields in all recordings.

### Changes in response properties

**Fig. 7.**
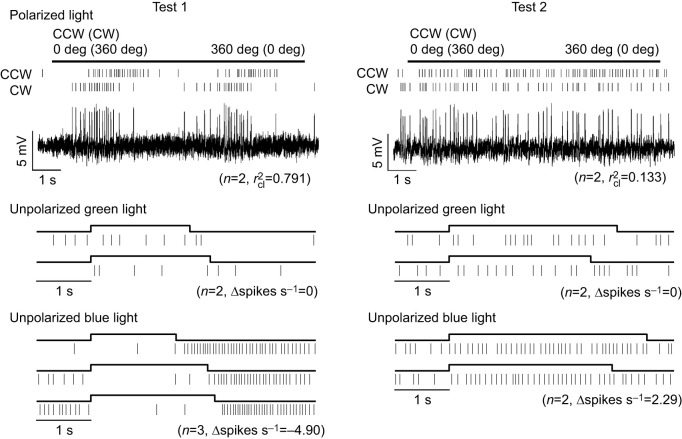
**Changes in neural response properties of a TL2a neuron.** Spike rasters are aligned to the onset of the zenithal light stimuli (straight line or square wave above the respective rasters). Waveforms were high-pass filtered for display purposes. Test 1 was performed at the beginning of the recording and test 2 responses were recorded roughly 30 min later.

Finally, we investigated changes in response properties that occurred in some neurons during the recording. Particularly prominent changes, including a response reversal, were observed in a TL2a neuron (TL2a_09 in Fig. 7 and Table S1). Responses to zenithal visual stimuli were tested twice, at the beginning of the recording and roughly 30 min later. The neuron showed an AoP response during the first test period, while no AoP response was observed during the second testing (top traces in Fig. 7, *r*_cl_^2^=0.791 in test 1 and *r*_cl_^2^=0.133 in test 2). The lack of AoP response during the second test was likely caused by the disappearance of inhibition at Φ_min_ (Table S1, the first Φ_max_ activity/amplitude=0.973 and the second Φ_max_ activity/amplitude=1.45). Similarly, the inhibitory response to unpolarized blue light spots changed to an excitatory response (bottom in Fig. 7, Δspikes s^−1^=−4.90 in test 1 and Δspikes s^−1^=2.29 in test 2). No response was observed during both test periods to unpolarized green light spot presentation (middle in Fig. 7).

In the recording of another TL2a neuron (TL2a_31), we tested zenithal AoP responses seven times (results of first, fourth and last testing in Table S1). AoP responses were stable during the first three test periods (∼10 min) but were not present during tests 4 and 5. AoP response returned in tests 6 and 7, roughly 5 min after the no response activities. Similarly to the TL2a_09 neuron, inhibition at Φ_min_ angle was not observed during tests 4 and 5 (Φ_max_ activity/amplitude=3.53 and 2.28). Responses to stationary light spots were not tested in this neuron.

Zenithal responses were, likewise, tested twice with a time interval >10 min in six other TL2a neurons and two TL3b neurons. AoP response properties of one TL2a neuron (TL2a_38) and the two TL3b neurons were stable over time, while those of five TL2a neurons differed between the first and the second test of stimuli (summarized in Table S1). Especially in the second test of a TL2a neuron (TL2a_20), we observed no AoP response due to the disappearance of inhibition at Φ_min_, similarly to the examples described above. Some unknown factors may have modulated the balance of excitation and inhibition in these neurons during the recording.

## DISCUSSION

In the insect brain, TL/R neurons constitute the input to a ring attractor network in the CX, resulting in heading direction coding and steering commands transmitted to thoracic motor centers (Green et al., 2019; Heinze and Homberg, 2007, 2009; Nguyen et al., 2021; Omoto et al., 2017; Rayshubskiy et al., 2020 preprint; Seelig and Jayaraman, 2013, 2015; Shiozaki and Kazama, 2017; Sun et al., 2017; Vitzthum et al., 2002). We have characterized receptive field properties of two subtypes of these neurons, TL2 and TL3, in the locust by using visual stimuli simulating celestial compass cues across the sky. These neurons have large receptive fields for skylight polarization. However, unlike postsynaptic compass neurons of the CX (Zittrell et al., 2020), only a minority of the recorded neurons showed coding for polarization angles that match sky polarization patterns for particular sun positions. TL2 and TL3 neurons, in addition, have complex, spatially partitioned excitatory and inhibitory subfields for small light spots. In most cases, these subfields were not located in line with the polarization tuning, revealing substantial mismatch in compass coding through both sky compass cues.

### Input neurons to the CX network

TL/R neurons convey multiple cues to different layers of the CBL. These cues help to establish the animal's spatial orientation, including sky compass signals, object and visual panorama information, and wind direction (el Jundi et al., 2015; Hardcastle et al., 2021; Heinze and Reppert, 2011; Kim et al., 2019; Okubo et al., 2020; Pegel et al., 2018). A connectome analysis of the *Drosophila* CX suggests hierarchical competition between R neuron subtypes, in which different cues influence heading direction coding to various degrees (Hulse et al., 2021).

Our recordings were confined to TL2a, TL2b and TL3 neurons innervating layers 2, 3 and 5 of the locust CBL, respectively (Fig. 2). We consider these cell types and CBL layers as the main polarization inputs to the locust CX. However, our sampling may be unintentionally biased toward specific cell types owing to limitations of intracellular recordings, such as a preference for larger diameter neurons. Previous studies showed that TL1 and TL4 neurons are, likewise, sensitive to celestial cues, although their responses are much less pronounced than those of TL2 and TL3 neurons (Pegel et al., 2018; Vitzthum et al., 2002). They innervate layer 1 (TL4) or all layers of the CBL (TL1) and, therefore, complement the main polarization inputs by TL2 and TL3. Based on morphological criteria, TL2 neurons likely correspond to R2 cells and TL3 neurons to R3 cells in *Drosophila* (Omoto et al., 2017). Like R2 and R3 cells, all TL2 and TL3 neurons appear to be GABAergic, further supporting similar polarization-sensitive input architectures to the ring attractor networks in both species. However, in contrast to TL3 neurons, R3 cells in the fly are not sensitive to the orientation of polarized light and thus might have lost sensitivity to the polarization pattern in the sky (Hardcastle et al., 2021).

### Cell type-specific responses to sky compass signals

All TL subtypes were responsive to light stimuli simulating polarization and direct sunlight across the sky but differed in physiological properties (Figs 3–5, Figs S1–S4). Similar cell type-specific trends were reported previously (Bockhorst and Homberg, 2015; Heinze et al., 2009; Pegel et al., 2018, 2019; Vitzthum et al., 2002) but were not systematically analyzed. The different inputs to the different CBL layers may allow for dynamic head direction coding depending on sky conditions. The AoP sensitivity of TL2 neurons was highly dependent on stimulus position, which corresponds to the highly varying degree of polarization in the sky and should result in relatively good performance in matching sky polarization patterns (Fig. 5). Therefore, signals from TL2 neurons might be particularly useful under clear sky conditions. In contrast, TL3 neurons do not cover the full range of AoP orientations at the zenith (Fig. 3G), which is disadvantageous to matching sky polarization patterns. However, most TL3 neurons showed uniform high AoP sensitivity across large parts of the dorsal visual field, and thus, their signals may be robust even under cloudy or hazy sky conditions.

### Comparison to the postsynaptic network

The large size and position of receptive fields for AoP sensitivity of TL neurons (Fig. 4, Figs S2–S4) were similar to those of downstream neurons studied in Zittrell et al. (2020). Heinze et al. (2009) reported that TL2 neurons have medium-sized, ipsilaterally-biased receptive fields relative to other CX neurons, but this conclusion is based on only a few tested stimulus positions. Vitzthum et al. (2002) and Heinze et al. (2009) showed that AoP responses in TL3 neurons are mediated by the ipsilateral eye only (monocular input) but did not distinguish between TL3a and TL3b subtypes. We reanalyzed the morphology of those neurons and found that at least two of their recordings were from TL3b neurons. As shown here, most TL3b neurons were equally sensitive to AoP from ipsi- and contralateral directions, suggesting that their monocular input source did not limit their receptive field sizes within the range tested.

The preferred AoP of TL neurons changed gradually within the 120 deg range around the zenith (Fig. 5, Figs S2–S4), again similarly to downstream neurons of the CX (Zittrell et al., 2020). Pattern matching between the AoP responses and sky polarization model yielded best matches to particular sun positions. However, judged by *P*-values obtained from bootstrapping procedure, the quality of the best match was good in only 10% of the cells compared to 74% in downstream neurons (Zittrell et al., 2020). Therefore, the matched filter properties in postsynaptic columnar neurons, such as CL1 or CPU types of the CX, are likely considerably refined by convergence and integration of synaptic input from appropriate TL neurons. In fact, E-PG neurons in *Drosophila* (equivalents to CL1 neurons) receive synaptic input from nearly all visually tuned R neurons (Hulse et al., 2021).

The two TL2 neurons with faithful polarization-matched filter quality also showed a good match in sun position coding through polarized and unpolarized light signaling. Although we did not find a distinct morphological difference between those two cells and the other TL2 neurons, there may be further subdivisions of TL2a and TL2b cell types based on physiological properties. Alternatively, response property changes observed in some recordings (Fig. 7 and Table S1) may contribute to differences in compass coding within the same cell type.

### Responsiveness to unpolarized light spots

The receptive fields for stationary unpolarized light spots comprised spatially distinct excitatory and inhibitory subfields in most neurons (Fig. 6, Figs S2–S4). This receptive field organization for visual stimuli is similar to that of R2 and R4d ring neurons in *Drosophila* (Seelig and Jayaraman, 2013). In the fly, inhibitory subfields are usually in close proximity to an excitatory area and partly surround an excitatory center, suggesting contrast enhancement for object detection similar to mechanisms in the mammalian visual cortex (Bonin et al., 2011). In the locust, however, both subfields were often spatially far apart and suggest spatial excitatory–inhibitory opponency across the sky, likely used to evaluate brightness contrast (Pegel et al., 2018; Pegel et al., 2019).

Some TL neurons possessed only excitatory or inhibitory fields for stationary light spots, and one TL2 neuron was completely unresponsive. In contrast, Pegel et al. (2018) found pronounced spatial opponency responses to rotating light spots in all TL2 neurons (7 recordings) compared to weaker responses in two TL3 neurons. A primary reason for this discrepancy may be the coarse grid of tested stimulus positions in our study. In addition, we used unpolarized light spots of 1.05 deg visual angle, which is closer to the apparent size of the sun (about 0.5 deg) but smaller than 16.3 deg light spots used by Pegel et al. (2018).

We found some differences between receptive field structures for unpolarized green and blue light. This difference may be critical for compass integration because the excitatory fields for blue light, in contrast to those for green light, were often far from the sun position estimated from AoP responses (Figs S2–S4). The response to green light originates from the main retina, while unpolarized blue light is detected by both the main retina and the dorsal rim area. In the main retina most photoreceptors co-express two types of opsins, a long wavelength (green)-absorbing type and a blue-absorbing type, while in the dorsal rim area, all photoreceptors express only a blue-absorbing opsin (Schmeling et al., 2014). It remains an open question how this receptive field difference affects solar azimuth detection.

### Changes in response properties

We observed response property changes in seven recordings out of 10 in which the response to zenithal stimuli was tested repeatedly over the course of the recording (Fig. 7 and Table S1). In three individuals, the AoP responses during the initial test were comprised of excitation at Φ_max_ and inhibition at Φ_min_, but inhibition at Φ_min_ was no longer detected during the following test. In one TL neuron, even a reverse response to unpolarized light spots was found. The balance of excitation and inhibition may have been modulated in these neurons by changes in the internal state of the animal, suggesting state-dependent processing of visual information in these neurons. Similar activity changes were found in *Drosophila* R neurons as epochs of elevated calcium activity visualized in glomeruli of the bulb, which were restricted to neurons in a specific glomerulus but not correlated with the activities of the upstream neuron in the same glomerulus (Sun et al., 2017). However, fluctuation in calcium activity was not reported when the activities to AoP stimuli were recorded as ensemble responses of R neuron populations (Hardcastle et al., 2021). Therefore, activity fluctuations may affect the dominance of TL/R neurons relative to others in the same layer and thereby control the output of the CBL/ellipsoid body to select visual features in a specific location.

The animal's behavioral state (rest, feeding, flying, etc.) may strongly affect the physiological properties of neurons, especially at higher level processing areas such as the CX. In this study and the work by Zittrell et al. (2020), all intracellular recordings were made from restrained, immobile animals. In monarch butterflies, extracellular recordings from neurons in tethered, flying animals demonstrated flight-induced changes in angular sensitivity of sun-compass neurons of the CX, likely induced by octopamine (Beetz et al., 2022). Therefore, in the locust, the neural activities of TL inputs and downstream cells may, likewise, change as the animal starts flying, and thus, the matched filter qualities of these cell types may improve or change in other ways to be explored.

Visual features of the sky are reliable compass cues owing to their persistent presence during navigation. Parallel channels for celestial cues as inputs to the CX are likely combined and refined by the compass network in the CX to yield a robust heading signal based on a combination of sky compass cues that eventually leads to accurate spatial orientation.

## Supplementary Material

10.1242/jexbio.243858_sup1 Supplementary information
