# Supplementary material for: Receptive field structures for two celestial compass cues at the input stage of the central complex in the locust brain
Source: J Exp Biol. 2022 Feb 23;225(4):jeb243858. doi: 10.1242/jeb.243858 (PMC10215807; doi:10.1242/jeb.243858)
Supplement: Supplementary information [file jexbio-225-243858-s1.pdf]

Supplementary Materials and Methods

Circular-linear correlation

To calculate the circular-linear correlation coefficient (r_{cl}), we used the function "circ_corrcl" in the "Circular Statistics Toolbox" of MATLAB (Berens, 2009). When a circular variable is α and a linear variable is x, this function defines the correlation coefficients $r_{sx} = c(\sin\alpha, x)$, $r_{cx} = c(\cos\alpha, x)$ and $r_{cs} = c(\sin\alpha, \cos\alpha)$, where $c(x, y)$ is the Pearson correlation coefficient. Then the circular-linear correlation coefficient r_{cl} is computed as follows;

$$r_{cl} = \sqrt{\frac{r_{cx}^2 + r_{sx}^2 - 2r_{cx}r_{sx}r_{cs}}{1 - r_{cs}^2}} \qquad (1)$$

To judge AoP responses, we used the square value of the coefficient (r_{cl}^2) because it follows a χ^2 distribution with two degrees of freedom (Berens, 2009).

Great-circle distance

Generally, for spherical coordinates of a given point α (azimuth α_1, elevation α_2) with $0° \leq \alpha_1 < 360°$ and $0° \leq \alpha_2 \leq 90°$ (Fig. 1A,C), the position vector $\vec{\alpha}$ is

$$\vec{\alpha} = \begin{pmatrix} \cos\alpha_1 \cdot \cos\alpha_2 \\ \cos\alpha_1 \cdot \sin\alpha_2 \\ \sin\alpha_1 \end{pmatrix} \qquad (2)$$

The great-circle distance θ between the points α and β is calculated using vector products as follows;

$$\theta = arccos(\vec{\alpha} \cdot \vec{\beta}) = arctan\frac{|\vec{\alpha} \times \vec{\beta}|}{\vec{\alpha} \cdot \vec{\beta}} \qquad (3)$$

Single-scattering Rayleigh model

We generated sky polarization patterns (angles and degrees of polarization) based on the single-scattering Rayleigh model (Strutt, 1871). The angle of polarization (AoP) at a given point of the sky is perpendicular to a great circle passing through the sun and the subject point. Thus, the vector of AoP is calculated as the cross vector product;

$$\overrightarrow{AoP} = \vec{s} \times \vec{p} \qquad (4)$$

where $\vec{s}$ and $\vec{p}$ are the position vectors of the sun and the subject point, respectively. The degree of polarization (DoP), or percent polarization, varies between 0 (for unpolarized light) and 1 (for completely polarized light). In the single-scattering

Journal of Experimental Biology • Supplementary information

Rayleigh model, the DoP is calculated as a function of the great-circle distance between the sun and the subject point;

$$DoP = \frac{1-\cos^2\theta}{1+\cos^2\theta} \qquad (5)$$

where θ is the great-circle distance between $\vec{s}$ and $\vec{p}$. The DoP reaches its maximum (= 1) when the great-circle distance is 90°.

Background activity (BA).

Spikes were counted per 1-s bins as background activity (BA) of the neurons (Fig. S1). We used all bins during the absence of stimulation and current injection for the analysis, except during 5 s after the light was turned off to exclude rebound responses. We sometimes observed spike rate changes lasting after light stimuli were turned off. Such long-lasting aftereffects were more frequently observed in TL3 than in TL2 neurons. However, as we did not have objective means to isolate these effects from spontaneous changes in BA, we used the whole recording fulfilling the criteria to avoid arbitrary omission of parts of the recording.

To evaluate BA characteristics of each cell type, we calculated the mean and Fano factor of spike counts per bin. Fano factor is the variance to mean ratio (variance/mean) of count data, commonly used to evaluate variability (Fano, 1947; Rajdl et al., 2020). That is because ideal count data follow a Poisson-distributed process where mean equals variance. When the Fano factor > 1 or < 1, BA is considered more fluctuating or more constant through the whole recording, respectively.

For statistical comparison of BA mean levels between cell types, we constructed a generalized linear model (GLM) of a gamma distribution by function “glm” in the “stats” package of R (R Core Team, 2021). The link function of GLM was “identity.” Response variables were BA mean of individuals, and fixed effects were cell types. The statistical significance of a fixed effect was tested by Wald test of an estimated coefficient (Faraway, 2016). In this method, the test static z is obtained by dividing the coefficient value by its s.e.. The distribution of z is approximated by a normal distribution to calculate the p value under the null hypothesis that the coefficient = 0.

Journal of Experimental Biology • Supplementary information

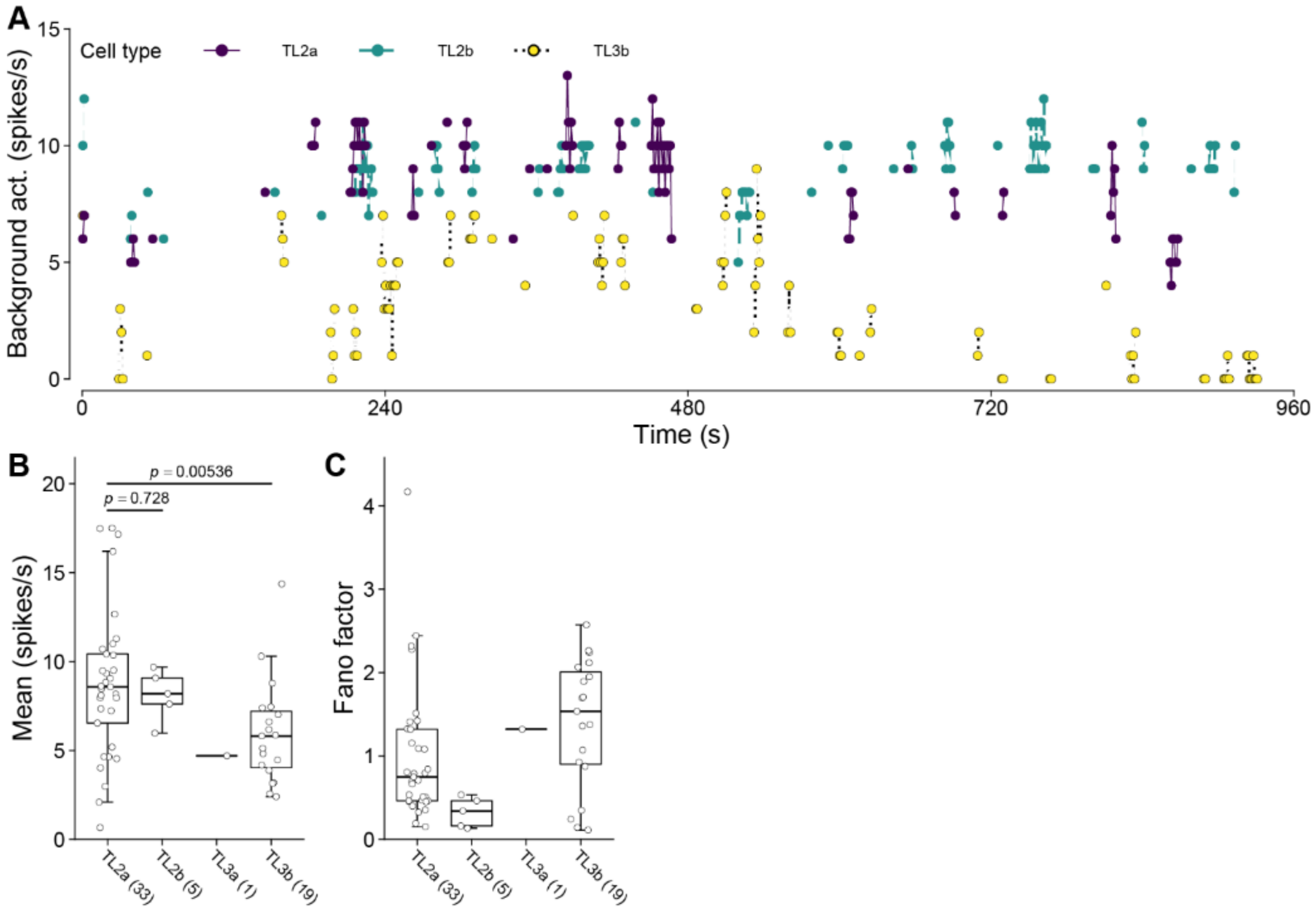

Fig. S1. Background activity (BA).

(A) BA examples of three single neurons: TL2a_03 (dark purple), TL2b_01 (green), and TL3b_04 (yellow) throughout the recordings (~ 16 min). Data points indicate spike counts per 1-s bins. Line-connected points are consecutive bins not interrupted by a stimulus or current injection.

(B,C) Box plots showing group data for each cell type. (A) variation of mean BA levels; (B) distribution of Fano factor (variance/mean) throughout the recordings. Eight recordings were excluded because only short parts (< 30 s) were available for BA analysis. The *p* values of Wald test of fixed effect coefficients are shown in A. TL2a cell type was defined as the baseline (control) to estimate the fixed effect coefficients of the GLM because the sample number was the largest. The only TL3a cell was excluded from the statistical test. The BA of each cell type showed several characteristics. TL2a and TL2b neurons shared similar BA levels (A), with median group activities of 8.6 and 8.2 spikes/s, respectively, and a significant difference was not detected (Wald test,

coefficient = −0.0640, z = −0.349, $p = 0.728$). In contrast, the median BA level of TL3b neurons was 5.8 spikes/s, which was significantly lower than that of TL2a neurons (coefficient = −2.77, z = −2.90, $p = 0.00536$). The only TL3a neuron showed a TL3b-like BA level (4.7 spikes/s). We also investigated the variability of BA throughout the recordings by calculating Fano factor, or variance to mean ratio, of spike counts per bin (B). The BA of TL2b neurons was highly constant throughout the recordings (group median of Fano factor < 0.5), in contrast to that of TL3b neurons whose BA fluctuated more strongly (group median of Fano factor > 1.5). Naturally, there were outliers from the general trend for each cell type. Especially in TL2a neurons, BA levels were more dependent on the individually recorded cell (A).

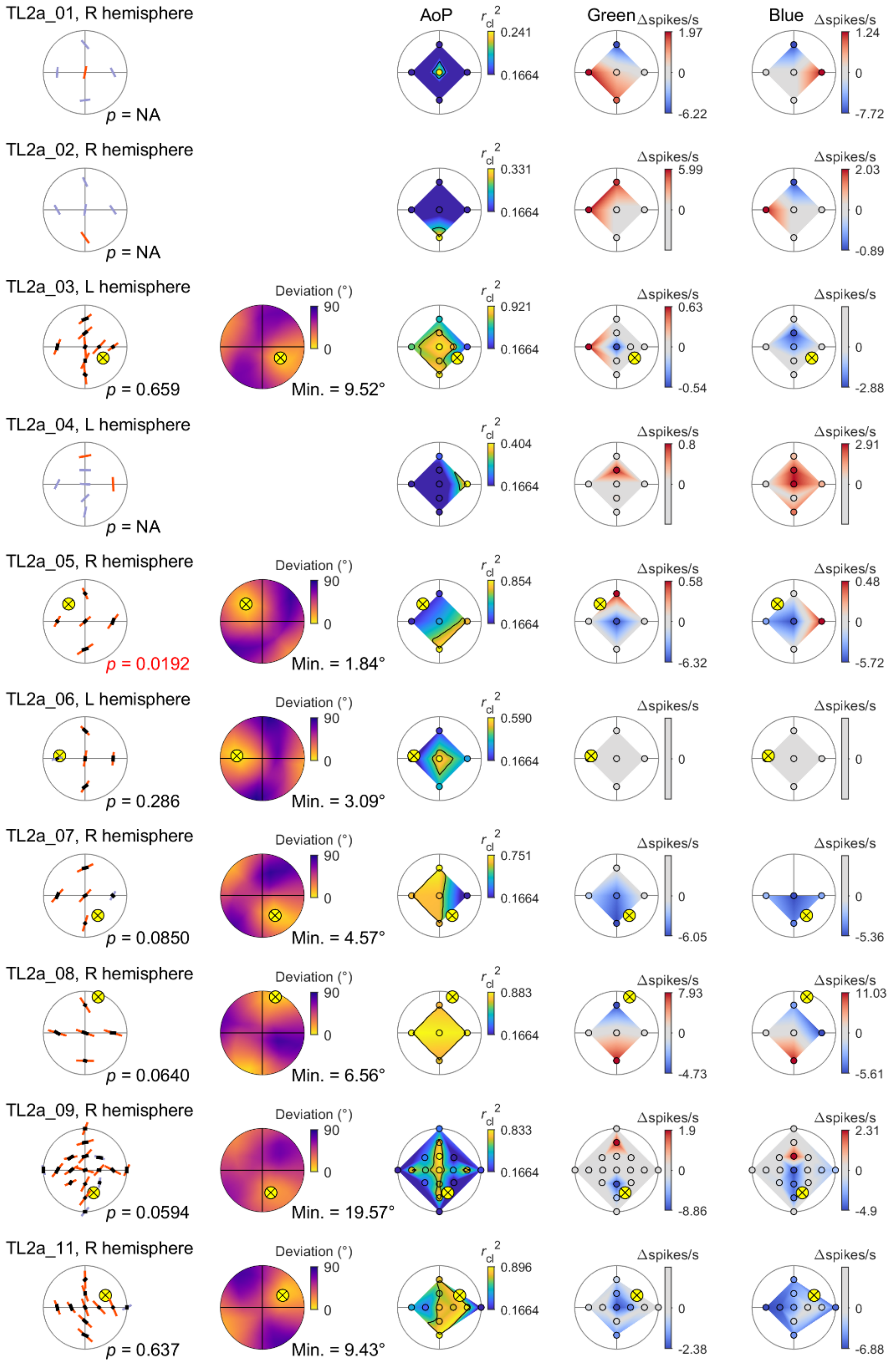

Journal of Experimental Biology • Supplementary information

Fig. S2. Receptive fields and AoP pattern fitting results (related to Figs. 4–6): TL2a neurons (ten out of 15 cells).

Each row shows the data from a single neuron. The information of cell type, ID, and brain hemisphere of its soma is indicated on the upper left corner of each row. All plots are top views on flattened sky hemispheres (see Fig. 1D for the coordinate system). Plots in column 1 indicate best matching sky polarization patterns and the corresponding sun positions, as shown in Fig. 5A. Plots in column 2 are related to the pattern matching analysis (Fig. 5), showing linearly interpolated pattern deviations between the AoP response pattern and sky polarization patterns generated by various solar coordinates. Values at the bottom indicate the minimum pattern deviations (best match) yielded from the solar coordinates indicated by a crossed yellow circle. Plots of column 3 indicate polarization sensitivity as shown in Fig. 4A, and plots of columns 4 and 5 indicate the receptive field organizations to unpolarized green light as in Fig. 6C and unpolarized blue light, respectively. Blank spaces are properties that were not measured in the respective neurons.

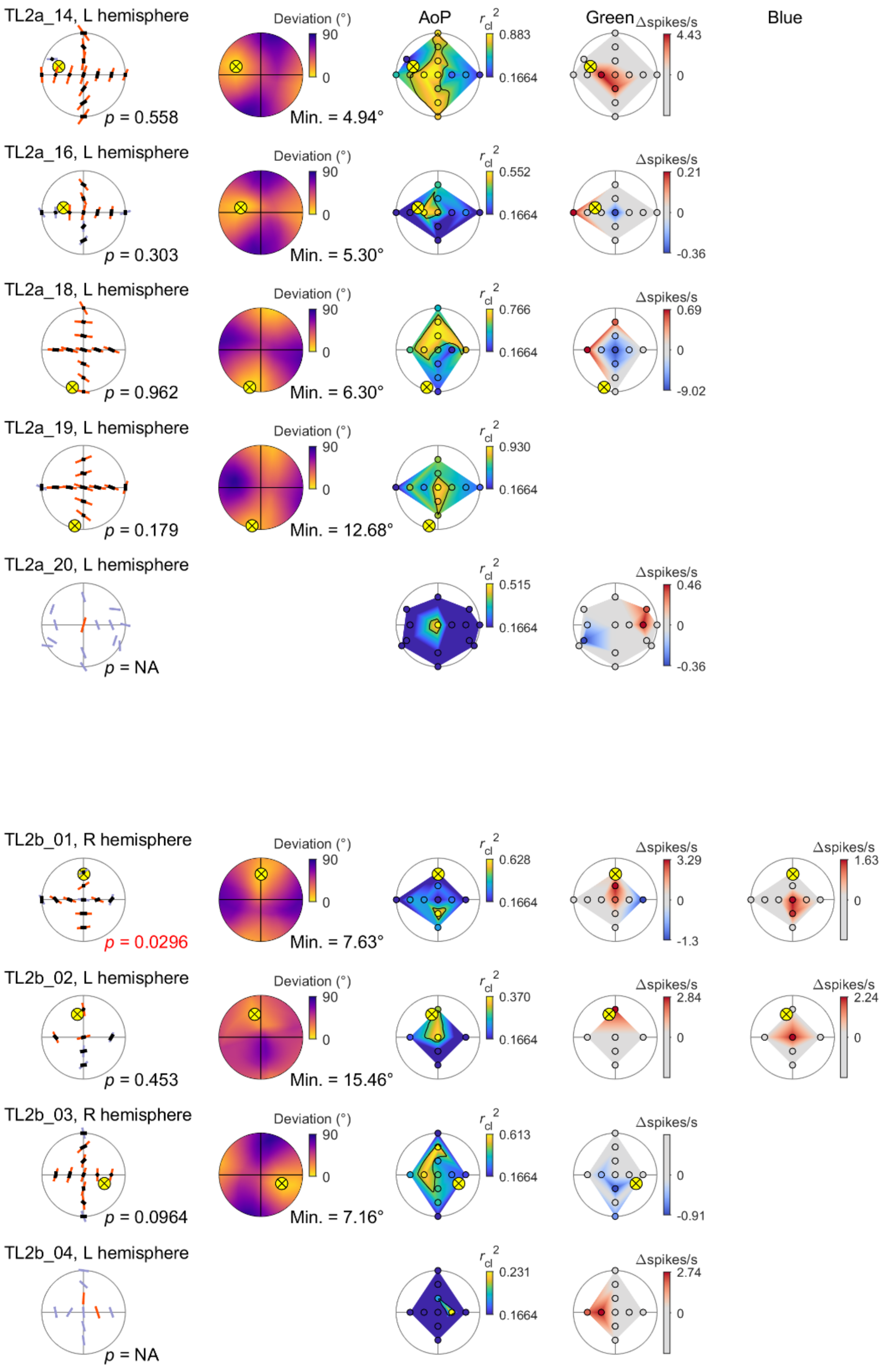

Fig. S3. Receptive fields and AoP pattern fitting results (related to Figs. 4–6): TL2a (five out of 15 cells) and TL2b neurons (four cells).

All plots are arranged as shown in Fig. S2.

Journal of Experimental Biology • Supplementary information

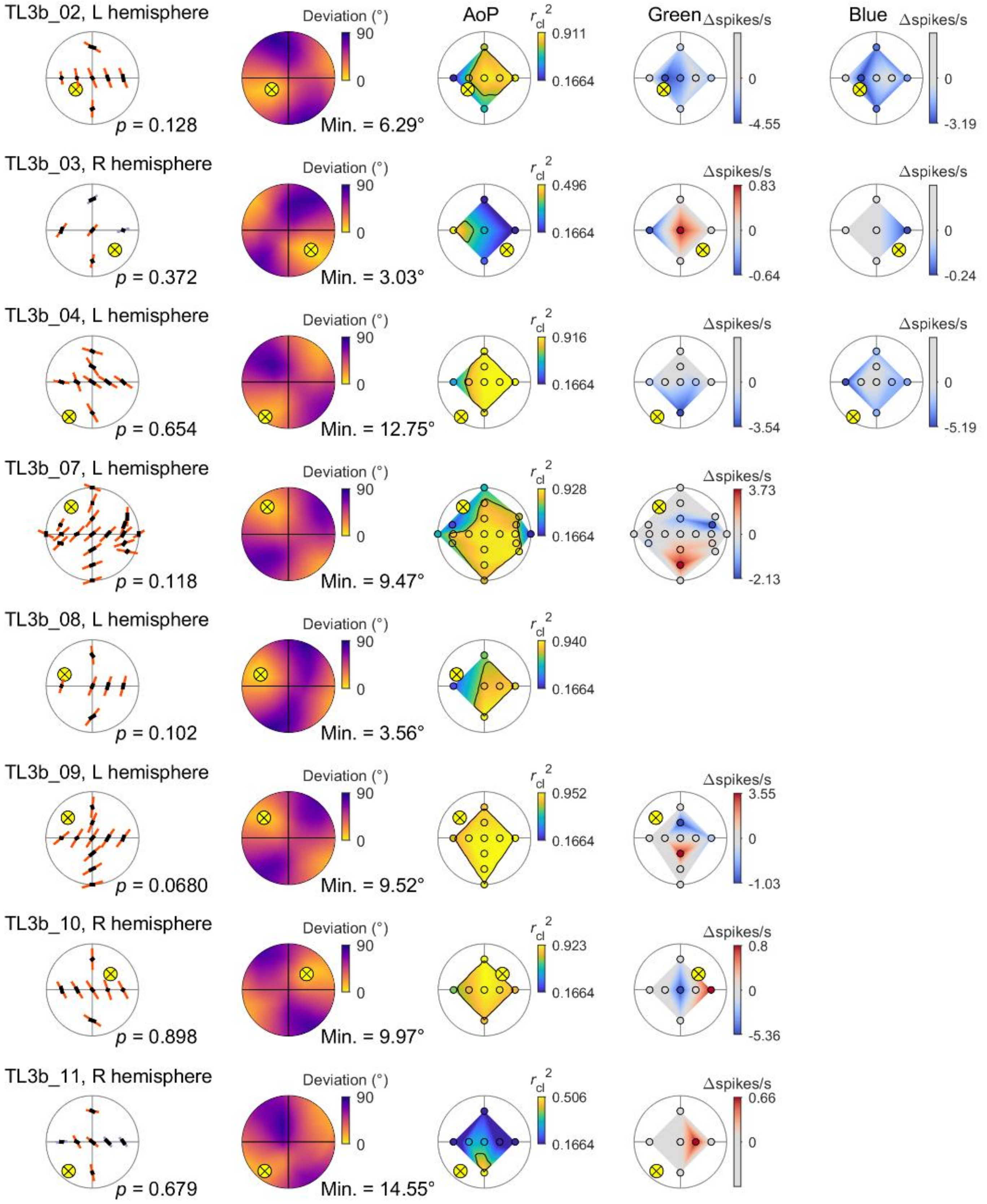

Fig. S4. Receptive fields and AoP pattern fitting results (related to Figs. 4–6): TL3b (eight cells).

All plots are arranged as shown in Fig. S2.

Journal of Experimental Biology • Supplementary information

Table S1. Changes in response properties to zenithal stimulation in TL neurons.

				AoP			Light spot (Δspikes/s)	
ID	Test #	Interval (s)	BA level (spikes/s)	r_{cl}^2	Φ_{max} (°)	Φ_{max} act./amp.	Green	Blue
TL2a_03	1	0	6.50	0.921	4.7	0.718	-0.54	-1.88
	2	610	7.00	0.612	150.4	0.638	NA	NA
TL2a_09	1	0	0.50	0.791	74.3	0.973	0.00	-4.90
	2	1720	4.00	0.133	75.5	1.446	0.00	2.29
TL2a_11	1	0	9.00	0.800	20.3	0.590	-2.38	-4.54
	2	990	11.67	0.748	43.2	0.255	-2.12	-1.02
TL2a_16	1	0	1.25	0.502	166.8	0.625	-0.36	NA
	2	910	0.00	NA	NA	NA	0.16	NA
TL2a_20	1	0	2.50	0.515	164.4	0.819	0.00	NA
	2	1470	9.08	0.013	78.9	3.375	NA	NA
TL2a_31	1	0	12.25	0.895	59.9	0.630	NA	NA
	4	660	9.43	0.051	133.8	3.533	NA	NA
	7	1060	15.41	0.828	58.6	0.587	NA	NA
TL2a_33	1	0	3.60	0.270	114.6	0.889	NA	NA
	2	850	2.15	0.772	98.9	1.023	NA	NA
TL2a_38	1	0	14.00	0.611	41.1	0.503	NA	NA
	2	680	6.75	0.868	39.3	0.700	NA	NA
TL3b_07	1	0	8.11	0.904	130.8	0.599	0.00	NA
	2	1050	6.00	0.892	133.3	0.769	0.11	NA
TL3b_19	1	0	6.00	0.895	27.6	0.665	NA	NA
	2	650	4.21	0.832	30.3	0.741	NA	NA

Mixed von Mises distributions were fitted to the spike activities during the AoP stimuli even if they were considered no response to AoP ($r_{cl}^2 < 0.1664$) in the same way as to AoP responses, to investigate Φ_{max} (°) and Φ_{max} activities/amplitude. Interval (s), measured from the last AoP stimulus in test #1 to the first AoP stimulus in test #2 (or #4, #7); BA level (spikes/s), BA mean used to calculate Φ_{max} act./amp.; NA, properties that were not calculated (AoP) or tested (light spot).

Journal of Experimental Biology • Supplementary information

References

Fano, U. (1947). Ionization yield of radiations. II. The fluctuations of the number of ions. *Phys. Rev.* **72**, 26–29.

Faraway, J. J. (2016). *Extending the linear model with R: generalized linear, mixed effects and nonparametric regression models*. 2nd ed. Boca Raton, FL, USA; London; New York, NY, USA: CRC Press.

Rajdl, K., Lansky, P. and Kostal, L. (2020). Fano factor: a potentially useful information. *Front. Comput. Neurosci.* **14**, 569049.
